# Variability of *Loa loa* microfilarial counts in successive blood smears and its potential implication in drug-related serious adverse events

**DOI:** 10.1186/s13071-024-06494-0

**Published:** 2024-11-08

**Authors:** Tristan M. Lepage, Jérémy T. Campillo, Frédéric Louya, Paul Bikita, François Missamou, Marlhand C. Hemilembolo, Sébastien D. S. Pion, Michel Boussinesq, Cédric B. Chesnais

**Affiliations:** 1 Service des Maladies Infectieuses et Tropicales, Hôpital La Colombière–CHU de Montpellier, Montpellier, France; 2 TransVIHMI, INSERM Unité 1175, Institut de Recherche pour le Développement (IRD), Université de Montpellier, Montpellier, France; 3 Programme National de Lutte contre l’Onchocercose, Direction de l’Épidémiologie et de la Lutte Contre la Maladie, Ministère de la Santé et de la Population, Brazzaville, Republic of Congo

**Keywords:** Loiasis, Diagnosis, Variability, Microfilaremia

## Abstract

**Background:**

The standard method to diagnose *Loa loa* infection and quantify microfilarial density (MFD) is the microscopic examination of calibrated thick blood smears (TBSs). In 1950, it was noticed that successive *L. loa* MFD samples from a single capillary puncture could exhibit up to 20% variation. Although loiasis treatment allocation is based on MFD to prevent serious adverse events (SAEs), data on this variability are scarce. There are also no guidelines supporting the collection and analysis of one or two TBSs.

**Methods:**

We assessed the variability of two successive *L. loa* MFD samples (MFD_1_ and MFD_2_), collected from 255 patients. We analyzed the influence of sex, age, weight, heart rate, arterial pressure, body temperature, and sampling time on MFD variability, as well the impact of MFD variability on MFD thresholds relevant to loiasis treatment protocols.

**Results:**

The MFD_2_ was found to have increased in 63% (1145/1826) of TBS pairs and to have decreased in 37% (681/1826) of TBS pairs. The MFD_2_ were on average 28% higher than the MFD_1_. These variations drove a total of 333 (17.4%) changes in MFD classes according to loiasis treatment protocol, including 210 (11.3%) class increases. TBSs generated from blood samples from subjects with lower MFD (1–1000 mf/ml) or lower mean arterial pressure (MAP; 55–80 mmHg), or from blood samples collected at an earlier hour time-point (10:00–10:59 a.m.) were more subject to MFD_2_ variability in a multivariate analysis. The MFD relative change was not constant over time for a given person.

**Conclusions:**

We observed a trend towards an increase in MFD_2_ with an important variability between samples that may impact loiasis treatment allocation. We suggest that systematically sampling at least two successive TBSs might allow better MFD assessments to prevent post-treatment SAEs. Further studies are needed to verify this variability in larger samples as well as confirm the potential explanatory variables identified.

**Graphical Abstract:**

**Figure:**
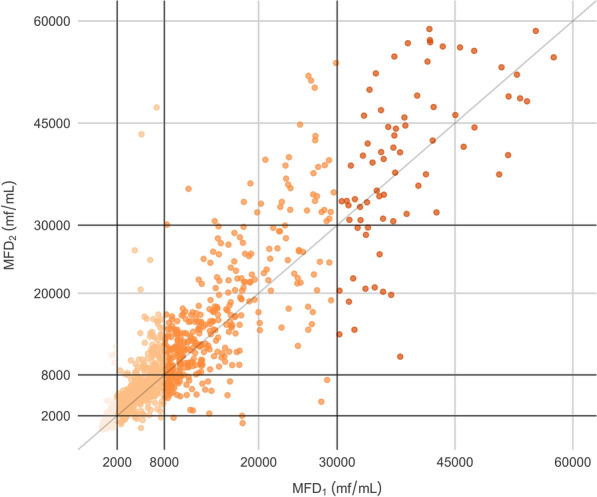

**Supplementary Information:**

The online version contains supplementary material available at 10.1186/s13071-024-06494-0.

## Background

Loiasis is a parasitic disease caused by the filarial nematode *Loa loa*. Transmitted from human to human by tabanids, it is exclusively present in Central Africa [1]. Adult stages live under the skin and in intermuscular fasciae, and female worms release embryos called microfilariae (mf), which circulate in the blood stream according to a diurnal periodicity [2]. In a number of rate cases, microfilarial density (MFD) can exceed 100,000 mf per milliliter of blood.

Three drugs can be used to treat subjects infected with *L. loa:* diethylcarbamazine (DEC), ivermectin (IVM) and albendazole [3]. Both DEC and IVM have a rapid and dramatic effect on mf, and subjects with high *L. loa* MFD may develop post-treatment serious adverse events (SAEs) [4] due to the embolization of capillaries by dead or paralyzed mf. Therefore, a standardized protocol [3] has been proposed to allocate treatment regimens according to patients’ MFD categories (1–2000, 2001–8000, 8001–30,000 and ≥ 30,000 mf/ml), with the aim to limit the risk of post-treatment SAEs.

The standard method to diagnose *L. loa* infection and quantify MFD is the microscopic examination of stained calibrated (usually 50 µl) thick blood smears (TBS) that have been prepared with peripheral blood collected by fingerprick. Several factors are known to impact MFD assessment. Sources of MFD variability include *L. loa* microfilarial diurnal periodicity [2, 5], day-to-day variability [6] and inter-reader variability [7], and all of these factors contribute to the difficulty in reliably assessing MFD, with a risk of misclassification between MFD categories leading to inappropriate treatment and ultimately SAEs.

In addition to microfilarial periodicity and day-to-day and inter-reader variability, it has also been noticed that successive TBSs from a single capillary puncture can yield significantly different results. In 1950, Kershaw performed five successive TBSs prepared from blood collected from two individuals and found that there was up to 20% variation in *L. loa* MFD [8]. To our knowledge, this is the only study to date that has investigated the variability of *L. loa* MFD on successive TBSs.

In clinical trials, two successive TBSs are usually collected from subjects, with averaged MFD taken into account [9, 10], whereas in routine care physicians most often estimate the MFD from a single TBS. Yet, to date, there are no clinical guidelines supporting the collection and analysis of one or two TBSs.

Here, we describe *L. loa* MFD variability between TBSs prepared using blood collected from two successive fingerpricks from 255 subjects. We also aimed to assess if this variability could lead to inappropriate treatment allocation [3], which would drive an increased risk of post-treatment SAEs. In addition, we evaluated the effect of potential explanatory factors on MFD variability, using clinical and demographic data.

## Methods

### Study population

Data were collected during a randomized controlled trial evaluating the safety and efficacy of levamisole in subjects with *L. loa* microfilaremia [9]. Briefly, 255 participants were recruited in 21 villages located within a 40-km radius around Sibiti, the capital town of the Lékoumou division (Republic of Congo), in a forested environment where loiasis is endemic.

### Parasitological assessment

Two successive 50-µl blood samples were collected from each trial participant 5 days before levamisole intake (D-5) and at each follow-up visit (2, 7 and 30 days after treatment [D2, D7, D30, respectively). Capillary blood was drawn between 10:00 a.m. and 4:00 p.m. from a single fingerprick, using a sterile lancet and a nonheparinized capillary. The two blood samples were spread on separate microscope slides to prepare TBSs (labeled MFD_1_ and MFD_2_ in the order of collection). The TBSs were then dried at room temperature, dehemoglobinized and stained with Giemsa within 24 h of collection. All *L. loa* mf present on the slides were counted using a microscope at 100-fold magnification. Counts were multiplied by 20 to express MFD in mf per milliliter. Each TBS pair was assessed independently by two experienced laboratory technicians.

### Statistical analyses

To exclude inter-reader variability, we compared MFD_1_ and MFD_2_ assessments made by a given microscopist. As a pair of TBS slides (referred to as MFD_1_ and MFD_2_) was prepared from the successive blood samples from each of the 255 participants at each of the four time-points, and as each pair was read by two microscopists, the expected number of pairs for comparison was 2040. Negative pairs (MFD_1_ and MFD_2_ = 0) and missing values (due to lost, broken, damaged or badly discolored slides) were excluded from the analysis. Ultimately, a total of 1826 pairs were analyzed.

#### Possible factors contributing to MFD variability

Demographic and clinical data were collected for each participant, including age, sex and body weight. Vital signs (mean arterial blood pressure [MAP], heart rate, body temperature) were measured on D-5 and on the D2 and D7 follow-up visits, but not on D30. Additional details on the procedure have been published elsewhere [9]. Explanatory variables were discretized as follows:

- Age: 18–40, 41–50, 51–60 and 61–70 years
- Weight: 40–55, 56–60, 61–65 and 66–85 kg
- Heart beats per minute (bpm): 50–65, 66–75, 76–85 and 86–115 bpm
- MAP: 55–80, 81–90, 91–100, 101–150 mmHg
- Body temperature: 34.3–36.3 °C, 36.4–36.5 °C, 36.6–36.7 °C and 36.8–37.8 °C
- MFD: 1–1000, 1001–3000, 3001–9000 and 9001–85,000 mf/ml
- Sample collection time: 10:00–10:59 a.m., 11:00–11:59 a.m., 12:00–12:59 p.m. and 13:00–13:59 p.m.

#### Descriptive and univariate analyses

MFD_1_ and MFD_2_ were compared using the Wilcoxon rank-signed test, and their geometric means (GM) were calculated. MFD absolute difference was defined as: MFD_2_ − MFD_1__._ The MFD ratio was defined as: MFD_2_/MFD_1_. Tables were constructed to compare the distribution of MFD_1_ and MFD_2_ in MFD classes (0–2000, 2001–8000, 8001–30000 and ≥ 30,000 mf/ml) as proposed by a previous loiasis treatment protocol [3]. The distribution of the arithmetic means of MFD_1_ and MFD_2_ (a metric often used in clinical trials on loiasis) was also compared to that of MFD_1_ and that of MFD_2_.

The MFD relative change between the two successive TBSs was defined as: ([MFD_2_ − MFD_1_]/MFD_1_) × 100. Positive relative changes (MFD_2_ ≥ MFD_1_) and negative relative changes (MFD_2_ < MFD_1_) were analyzed separately. MFD relative changes among categorical variables (sex, age class, weight class, heart rate class, MAP class, body temperature class, MFD_1_ class, microscopist, collection hours and trial time-point) were compared using nonparametric tests for paired data (Wilcoxon rank-sum test and Kruskal–Wallis test in two- and four-level variables, respectively). In the case of a significant difference, Cuzick’s test was performed to assess the presence of a trend.

Descriptive bivariate statistics tables were generated to compare the characteristics of subjects with marked relative differences between MFD_1_ and MFD_2_ (beyond the MFD_1_ arithmetic mean + 1 standard deviation [SD] and beyond the MFD_1_ arithmetic mean + 2 SD) with the rest of the study population (control group), focusing on MFD_2_ increase (Table 2) and MFD_2_ decrease (Table 3). MFD relative change proportions among categorical explanatory variables were assessed with Pearson’s Chi-squared test and Fisher’s exact test, depending on sample size. Continuous variables (age, weight, heart rate, MAP, body temperature and MFD) were evaluated with the Wilcoxon rank-sum test.

#### Stability of individuals’ MFD change over time

We analyzed whether the sign of the difference between MFD_1_ and MFD_2_ was consistent at other time-points in all individuals. Stable MFD (MFD_2_ = MFD_1_) was arbitrarily defined as MFD_2_/MFD_1_ = 0.8–1.2. MFDs assessed by the two different microscopists were analyzed separately. Kappa’s coefficient agreements and McNemar’s tests were performed to assess the stability of distributions between two time-points.

#### Multivariate analyses

Since the outcomes are count data, MFD relative change was discretized into two categories (above and below the upper quartile) to perform a binomial logistic regression. We also performed a regression comparing TBSs with a class increase to TBSs bearing no class change, using MFD categories proposed in a previous loiasis treatment protocol [3]. Because vital signs were not collected at the D30 follow-up visit (representing > 25% missing data), we excluded D30 data from the regression. Saturated logistic regression was performed, adjusted for all covariates. Interactions of interest (age and MAP; age and MFD; sex and weight; sex and MAP; MAP and heart rate; body temperature and MFD) were checked using likelihood ratio tests. A procedure of Akaike information criterion (AIC)-based descending selection was then applied to select the final model. Finally, Wald’s test was applied to assess overall significance for categorical variables.

Analyses were performed in the R-Cran statistical environment, version 4.2.0 ® Foundation for Statistical Computing, Vienna, Austria) and Stata v18 (2023) software (StataCorp LLC, College Station, TX, USA).

## Results

### Baseline characteristics

A total of 1826 TBS pairs from 255 patients (70% males) with a mean age of 48 years were included in the analysis. The mean MFD was 7443 mf/ml, with 32% of all MFD_1_ and MFD_2_ having MFD < 1000 mf/ml and 23% having MFD > 9001 mf/ml. At the time of sample collection, no patients were febrile (maximum body temperature: 37.8 °C). The mean heart rate and mean MAP were 74 bpm and 93 mmHg, respectively, and blood for 40% of TBS was collected between 11:00 a.m. and 11:59 a.m.

### Comparison of MFD_1_ and MFD_2_

**Fig. 1:**
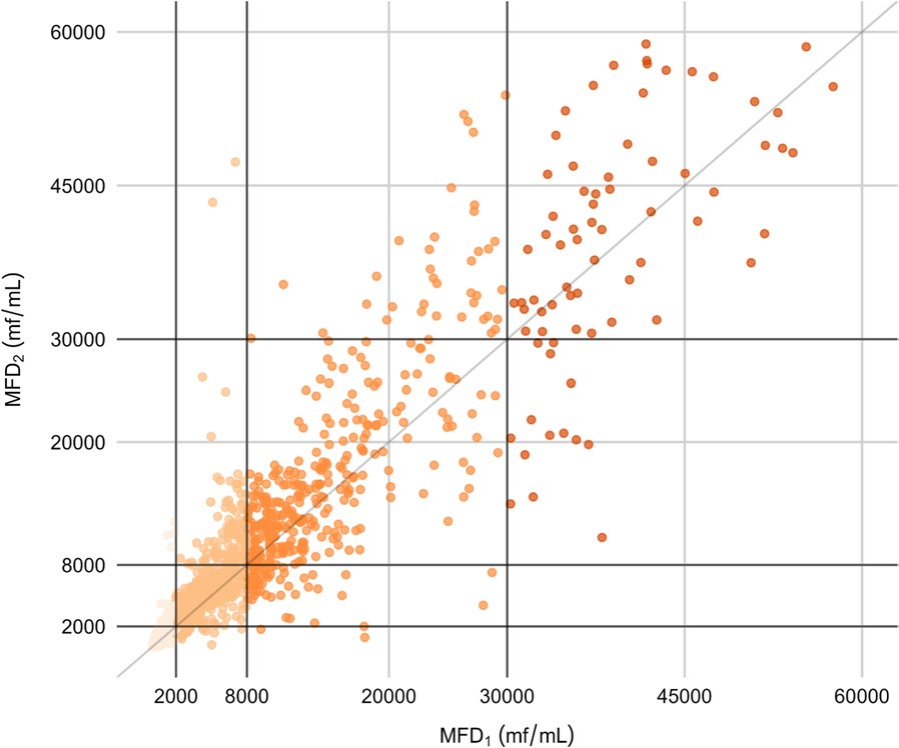
Scatter plot of MFD_2_ against MFD_1_. Horizontal and vertical black lines represent MFD cut-offs of 2000, 8000 and 30,000 mf/ml defined in loiasis treatment protocol [4], allowing graphic visualization of individuals changing classes between MFD_1_ and MFD_2_ classes. Colors represent the MFD_1_ class. MFD_1_ and MFD_2_, microfilarial density of two thick blood smears generated from two successive blood samples; mf, microfilariae

The MFD was visualized in a scatter plot of MFD_2_ against MFD_1_ (Fig. 1). Values of MFD_2_ greater than those of MFD_1_ are represented above the dark identity line (*x* = *y*), while values of MFD_2_ less than those of MFD_1_ are below. Overall, the MFD_2_ of 63% (1145/1826) of TBSs exhibited were determined to an increased MFD, while only 37% (681/1826) exhibited a decrease, relative to MFD_1_.

**Fig. 2:**
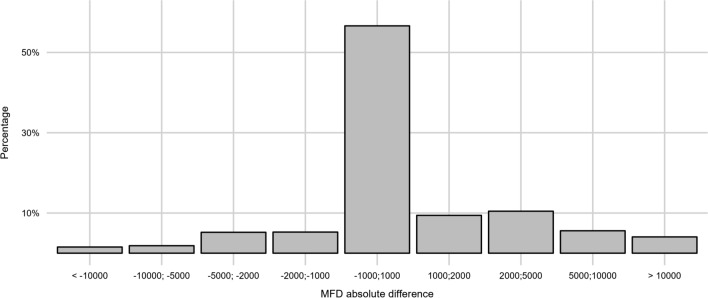
Bar chart of the distribution of the MFD absolute difference (MFD_2_ − MFD_1_) categories in percentages. MFD, microfilarial density; MFD_1_ and MFD_2_, microfilarial density of two thick blood smears generated from two successive blood samples

The distribution of the MFD absolute differences is presented in Fig. 2. The MFD of 56.6% TBS pairs differed by − 1000 to + 1000 mf/ml, while 29.5% and 9.6% of TBS pairs had an MFD absolute difference that surpassed + 1000 and + 5000 mf/ml, respectively. In comparison, 13.9% and 3.4% of TBS pairs had an MFD absolute difference the fell below − 1000 and − 5000 mf/l, respectively. The arithmetic mean of the MFD absolute differences was 967.7 mf/ml.

Overall, the GM of the MFD_2_ was higher than that of the MFD_1_ (2570 vs 2335 mf/ml), with a significantly different distribution (Wilcoxon signed-rank test, Z = −11.2, *P* < 0.001; Additional file 1: Table S1). The arithmetic mean of the MFD ratio (defined as MFD_2_/MFD_1_) was 1.28. The higher value of MFD_2_ compared to MFD_1_ was consistently observed across all subgroups (Additional file 1: Table S1).

### Transition evaluation over time at an individual-level

A transition evaluation was conducted to determine whether individuals with an MFD_2_ above, below or equivalent to MFD_1_ at any time-point would show the same trend at other time-points. Kappa’s coefficients were < 0.1, and no McNemar tests were significant between all time-points (Additional file 1: Tables S2–S4). The MFD relative change in our samples were therefore not constant over time for a given person, as individual results at one time-point were not correlated to those at other time-points.

### MFD relative change

Overall, mean MFD relative change was + 61% in TBSs with an MFD_2_ increase, whereas it was − 29% in TBSs with an MFD_2_ decrease (Table 1). Among several subgroups (age, weight, MAP, body temperature, MFD_1_ class or sample collection time-point), some categories exhibited significantly different relative changes. Therefore, we investigated how these variables could explain the differences between MFD_1_ and MFD_2_.

**Table 1:** Comparison of microfilarial density (MFD) relative change in MFD_2_ with increased or decreased MFD

Characteristic	MFD_2_ increase			MFD_2_ decrease		
	*N*	Relative change, mean (SD)	*P* value^a^	*N*	Relative change, mean (SD)	*P* value^a^
*Total*	1145	61% (89)		681	− 29% (21)	
Sex						
Male	806	62% (83)	0.042	471	− 29% (22)	0.283
Female	339	60% (100)		210	− 30% (20)	
Age class (years)						
18–40	348	57% (76)	0.602	206	− 30% (20)	< 0.001 *P* trend 0.103
41–50	245	54% (62)		154	− 24% (20)	
51–60	323	68% (98)		203	− 29% (21)	
61–70	229	66% (112)		118	− 35% (23)	
Weight class (kg)						
40–55	442	58% (98)	0.003 *P* trend 0.007	307	− 29% (21)	0.495
56–60	336	61% (70)		174	− 29% (22)	
61–65	167	61% (95)		123	− 29% (20)	
66–85	200	69% (87)		77	− 27% (24)	
Heart rate class (bpm)						
50–65	211	62% (85)	0.519	128	− 30% (22)	0.671
66–75	264	51% (67)		148	− 28% (20)	
76–85	223	64% (124)		153	− 30% (21)	
86–115	102	58% (75)		112	− 28% (21)	
MAP class (mmHg)						
55–80	167	55% (64)	0.768	110	− 33% (21)	0.010 *P* trend < 0.001
81–90	221	66% (119)		138	− 33% (24)	
91–100	205	64% (107)		151	− 27% (18)	
101–150	209	46% (50)		144	− 25% (19)	
Body temperature class						
34.3–36.3 °C	249	62% (117)	0.045 *P* trend 0.014	177	− 31% (20)	0.013 *P* trend 0.977
36.4–36.5 °C	161	47% (56)		100	− 24% (19)	
36.6–36.7 °C	193	56% (75)		138	− 28% (22)	
36.8–37.8 °C	199	64% (92)		128	− 32% (22)	
MFD_1_ class (mf/ml)						
1–1000	369	91% (117)	< 0.001 *P* trend < 0.001	213	− 37% (22)	< 0.001 *P* trend < 0.001
1001–3000	223	56% (84)		144	− 28% (20)	
3001–9000	298	52% (73)		150	− 23% (18)	
9001–85,000	255	33% (30)		174	− 25% (20)	
Microscopist						
Microscopist 1	577	67% (99)	0.028	340	− 29% (21)	0.609
Microscopist 2	568	55% (76)		341	− 29% (22)	
Collection time (hour of day)						
10:00–10:59 a.m.	345	73% (106)	0.059	149	− 27% (20)	0.098
11:00–11:59 a.m.	414	56% (88)		317	− 31% (22)	
12:00–12:59 p.m.	292	54% (65)		175	− 28% (21)	
1:00–1:59 p.m.	94	64% (82)		40	− 26% (26)	
Collection time-point (day of study)^b^						
D-5	273	64% (109)	0.010 *P* trend 0.027	197	− 30% (21)	0.454
D2	260	49% (61)		195	− 29% (20)	
D7	284	63% (98)		159	− 28% (23)	
D30	328	67% (78)		130	− 28% (22)	

*bpm* Beats per minute, *MAP* mean arterial pressure, *mf* microfilariae, *MFD* microfilarial density, *MFD*_*1*_, *MFD*_*2*_ microfilarial density of two thick blood smears generated from two successive blood samples, *SD* standard deviation

^a^Kruskal–Wallis test, Wilcoxon rank-sum test, Cuzick’s test for trend (*P* trend)

^b^Blood samples were collected 5 days before levamisole intake (D-5) and at each follow-up visit (2, 7 and 30 days after treatment [D2, D7, D30, respectively])

The lowest MAP category (55–80 mmHg) was overrepresented in TBSs with an MFD_2_ increase beyond 2o SD, compared to the control group (41% vs 27%; Chi-squared test, χ^2^ = 8.63, df = 3, *P* = 0.035; Table 2). This overrepresentation was also observed in TBSs with an MFD_2_ decrease > 2 SD, compared to the control group (60% vs 24%; Chi-squared test, χ^2^ = 17.36, df = 3, *P* < 0.001, Table 3).

**Table 2:** Characteristics of thick blood smear pairs with an MFD_2_ relative increase of > 1 and > 2 standard deviations

Characteristic	Relative change < 1 SD, *N* = 1059	Relative change > 1 SD, *N* = 86	*P* value^a^	Relative change < 2 SD, *N* = 1098	Relative change > 2 SD, *N* = 47	*P* value^b^
Sex, *n* (%)			0.720			0.532
Male	744 (70%)	62 (72%)		771 (70%)	35 (74%)	
Female	315 (30%)	24 (28%)		327 (30%)	12 (26%)	
Age, mean (SD)	48 (13)	50 (12)	0.165	48 (13)	51 (12)	0.160
Age class (years), *n* (%)			0.462			0.386
18–40	327 (31%)	21 (24%)		337 (31%)	11 (23%)	
41–50	227 (21%)	18 (21%)		237 (22%)	8 (17%)	
51–60	293 (28%)	30 (35%)		305 (28%)	18 (38%)	
61–70	212 (20%)	17 (20%)		219 (20%)	10 (21%)	
Weight (kg), mean (SD)	58 (8)	60 (10)	0.370	58 (8)	59 (10)	0.741
Weight class (kg), *n *(%)			0.795			0.206
40–55	310 (29%)	26 (30%)		326 (30%)	10 (21%)	
56–60	156 (15%)	11 (13%)		162 (15%)	5 (11%)	
61–65	182 (17%)	18 (21%)		187 (17%)	13 (28%)	
66–85	411 (39%)	31 (36%)		423 (39%)	19 (40%)	
Heart rate (bpm), mean (SD)	73 (11)	74 (12)	0.898	73 (11)	74 (11)	0.822
Heart rate class (bpm), *n* (%)			0.492			0.828
50–65	251 (34%)	13 (25%)		257 (33%)	7 (26%)	
66–75	209 (28%)	14 (27%)		214 (28%)	9 (33%)	
76–85	93 (12%)	9 (17%)		98 (13%)	4 (15%)	
86–115	195 (26%)	16 (31%)		204 (26%)	7 (26%)	
MAP (mmHg), mean (SD)	93 (15)	90 (11)	0.427	93 (15)	88 (9)	0.165
MAP class (mm Hg), *n* (%)			0.167			0.035
55–80	207 (28%)	14 (27%)		210 (27%)	11 (41%)	
81–90	186 (25%)	19 (37%)		195 (25%)	10 (37%)	
91–100	156 (21%)	11 (21%)		162 (21%)	5 (19%)	
101–150	201 (27%)	8 (15%)		208 (27%)	1 (3.7%)	
Body temperature (°C), mean (SD)	36.53 (0.40)	36.51 (0.45)	0.743	36.53 (0.40)	36.46 (0.50)	0.659
Body temperature class, *n* (%)			0.622			0.461
34.3–36.3 °C	154 (21%)	7 (13%)		158 (20%)	3 (11%)	
36.4–36.5 °C	179 (24%)	14 (27%)		188 (24%)	5 (19%)	
36.6–36.7 °C	184 (25%)	15 (29%)		191 (25%)	8 (30%)	
36.8–37.8 °C	233 (31%)	16 (31%)		238 (31%)	11 (41%)	
MFD_1_ (mf/ml), mean (SD)	7480 (11,111)	1448 (3294)	< 0.001	7282 (10,990)	1071 (2035)	< 0.001
MFD_1_ class (mf/ml), *n *(%)			< 0.001			< 0.001
1–1000	213 (20%)	10 (12%)		218 (20%)	5 (11%)	
1001–3000	287 (27%)	11 (13%)		292 (27%)	6 (13%)	
3001–9000	253 (24%)	2 (2.3%)		255 (23%)	0 (0%)	
9001–85000	306 (29%)	63 (73%)		333 (30%)	36 (77%)	
Microscopist, *n* (%)			0.412			0.113
Microscopist 1	530 (50%)	47 (55%)		548 (50%)	29 (62%)	
Microscopist 2	529 (50%)	39 (45%)		550 (50%)	18 (38%)	
Collection time (hour of day), *n* (%)			0.210			0.049
10:00–10:59 a.m.	311 (29%)	34 (40%)		323 (29%)	22 (47%)	
11:00–11:59 a.m.	388 (37%)	26 (30%)		403 (37%)	11 (23%)	
12:00–12:59 p.m.	274 (26%)	18 (21%)		283 (26%)	9 (19%)	
1:00–1:59 p.m.	86 (8.1%)	8 (9.3%)		89 (8.1%)	5 (11%)	
Collection time-point, day of study, *n* (%)^c^			0.125			0.179
D-5	256 (24%)	17 (20%)		262 (24%)	11 (23%)	
D2	247 (23%)	13 (15%)		255 (23%)	5 (11%)	
D7	260 (25%)	24 (28%)		271 (25%)	13 (28%)	
D30	296 (28%)	32 (37%)		310 (28%)	18 (38%)	

Values for relative change are presented as the number of thick blood slides with the percentage (*n*/*N* × 100) presented in parentheses, unless otherwise indicated

*bpm* Beats per minute, *MAP* mean arterial pressure, *mf* microfilariae, *MFD* microfilarial density, *MFD*_*1*_, *MFD*_*2*_ microfilarial density of two thick blood smears generated from two successive blood samples, *SD* standard deviation

^a^Pearson’s Chi-squared test, Wilcoxon rank-sum test

^b^Pearson’s Chi-squared test, Fisher’s exact test, Wilcoxon rank-sum test

^c^Blood samples were collected 5 days before levamisole intake (D-5) and at each follow-up visit (2, 7 and 30 days after treatment [D2, D7, D30, respectively])

**Table 3:** Characteristics of thick blood smear pairs with an MFD_2_ decrease of < 1 and < 2 standard deviations

Characteristic	Relative change < 1 SD, *N* = 566	Relative change > 1 SD, *N* = 115	*P* value^a^	Relative change < 2 SD, *N* = 649	Relative change > 2 SD, *N* = 32	*P* value^b^
Sex, *n* (%)			0.746			0.021
Male	390 (69%)	81 (70%)		443 (68%)	28 (88%)	
Female	176 (31%)	34 (30%)		206 (32%)	4 (12%)	
Age, mean (SD)	48 (12)	49 (13)	0.407	48 (13)	49 (14)	0.591
Age class (years), *n* (%)			0.074			0.639
18–40	172 (30%)	34 (30%)		198 (31%)	8 (25%)	
41–50	136 (24%)	18 (16%)		148 (23%)	6 (19%)	
51–60	168 (30%)	35 (30%)		193 (30%)	10 (31%)	
61–70	90 (16%)	28 (24%)		110 (17%)	8 (25%)	
Weight (kg), mean (SD)	57 (8)	57 (9)	0.588	57 (8)	58 (7)	0.408
Weight class (kg), *n* (%)			0.958			0.564
40–55	145 (26%)	29 (25%)		163 (25%)	11 (34%)	
56–60	104 (18%)	19 (17%)		117 (18%)	6 (19%)	
61–65	63 (11%)	14 (12%)		73 (11%)	4 (12%)	
66–85	254 (45%)	53 (46%)		296 (46%)	11 (34%)	
Heart rate (bpm), mean (SD)	75 (12)	75 (14)	0.825	75 (12)	77 (15)	0.758
Heart rate class (bpm), *n* (%)			0.769			> 0.999
50–65	128 (28%)	20 (23%)		141 (27%)	7 (28%)	
66–75	128 (28%)	25 (29%)		146 (28%)	7 (28%)	
76–85	93 (20%)	19 (22%)		107 (21%)	5 (20%)	
86–115	105 (23%)	23 (26%)		122 (24%)	6 (24%)	
MAP (mmHg), mean (SD)	94 (14)	88 (12)	< 0.001	93 (14)	88 (10)	0.077
MAP class (mmHg), *n* (%)			0.007			< 0.001
55–80	108 (24%)	30 (34%)		123 (24%)	15 (60%)	
81–90	135 (30%)	16 (18%)		149 (29%)	2 (8.0%)	
91–100	85 (19%)	25 (28%)		106 (20%)	4 (16%)	
101–150	127 (28%)	17 (19%)		140 (27%)	4 (16%)	
Body temperature (°C), mean (SD)	36.46 (0.46)	36.56 (0.48)	0.055	36.47 (0.46)	36.59 (0.43)	0.147
Body temperature class, *n* (%)			0.090			0.260
34.3–36.3 °C	89 (20%)	11 (12%)		98 (19%)	2 (8.0%)	
36.4–36.5 °C	115 (25%)	23 (26%)		129 (25%)	9 (36%)	
36.6–36.7 °C	99 (22%)	29 (33%)		120 (23%)	8 (32%)	
36.8–37.8 °C	152 (33%)	25 (28%)		171 (33%)	6 (24%)	
MFD_1_ (mf/ml), mean (SD)	8761 (13,717)	5093 (9311)	< 0.001	8283 (13,331)	5259 (8082)	0.018
MFD_1_ class (mf/ml), *n* (%)			< 0.001			0.033
1–1000	119 (21%)	25 (22%)		140 (22%)	4 (12%)	
1001–3000	133 (23%)	17 (15%)		147 (23%)	3 (9.4%)	
3001–9000	154 (27%)	20 (17%)		166 (26%)	8 (25%)	
9001–85,000	160 (28%)	53 (46%)		196 (30%)	17 (53%)	
Microscopist, *n* (%)			0.366			0.993
Microscopist 1	287 (51%)	53 (46%)		324 (50%)	16 (50%)	
Microscopist 2	279 (49%)	62 (54%)		325 (50%)	16 (50%)	
Collection time (hour of day), *n* (%)			0.507			0.157
10:00–10:59 a.m.	129 (23%)	20 (17%)		145 (22%)	4 (12%)	
11:00–11:59 a.m.	257 (45%)	60 (52%)		299 (46%)	18 (56%)	
12:00–12:59 p.m.	146 (26%)	29 (25%)		169 (26%)	6 (19%)	
1:00–1:59 p.m.	34 (6.0%)	6 (5.2%)		36 (5.5%)	4 (12%)	
Collection time-point, day of study, *n* (%)^c^			0.404			0.392
D-5	161 (28%)	36 (31%)		190 (29%)	7 (22%)	
D2	169 (30%)	26 (23%)		188 (29%)	7 (22%)	
D7	132 (23%)	27 (23%)		148 (23%)	11 (34%)	
D30	104 (18%)	26 (23%)		123 (19%)	7 (22%)	

Values for relative change are presented as the number of thick blood slides with the percentage (*n*/*N* × 100) presented in parentheses, unless otherwise indicated

*bpm* Beats per minute, *MAP* mean arterial pressure, *mf* microfilariae, *MFD* microfilarial density, *MFD*_*1*_, *MFD*_*2*_ microfilarial density of two thick blood smears generated from two successive blood samples, *SD* standard deviation

^a^Pearson’s Chi-squared test, Wilcoxon rank-sum test

^b^Pearson’s Chi-squared test, Fisher’s exact test, Wilcoxon rank-sum test

^c^Blood samples were collected 5 days before levamisole intake (D-5) and at each follow-up visit (2, 7 and 30 days after treatment [D2, D7, D30, respectively])

The mean MFD was lower among subjects whose TBSs showed an MFD relative change > 1 SD, both in MFD_2_ increase (1448 vs 7480 mf/ml; Wilcoxon rank-sum test, U_(1145)_ = 72726, Z = 9.22, *P* < 0.001; Table 2) and MFD_2_ decrease (5093 vs 8761 mf/ml; Wilcoxon rank-sum test, U_(681)_ = 24393 , Z = 4.24, *P* < 0.001; Table 3), and that trend was also observed with MFD relative change > 2 SD.

Finally, an MFD_2_ increase > 2 SD was significantly more frequent among TBSs sampled between 10:00 a.m. and 10:59 a.m. (47% vs 29%; Pearson’s chi-squared test, χ^2^ = 7.86, df = 3, *P* = 0.049; Table 2), and an MFD_2_ decrease > 2 SD was significantly more frequent in males than in the control group (88% vs 68%; Pearson's chi-squared test, χ^2^ = 5.29, df = 1, *P* = 0.021; Table 3).

### Multivariate analyses

A binomial logistic regression was conducted to study the effect of adjusted explanatory variables on the variation between MFD_1_ and MFD_2_. The final models (according to AIC-based descending selection) are presented in the right columns of Table 4. In both final models for MFD_2_ increase and decrease, TBSs in the lowest MFD classes (1–1000 mf/ml) were subject to more variation (odds ratio [OR] 2.39, *P* < 0.001 and OR 1.72, *P* = 0.049, respectively) than in TBSs with an MFD ≥ 9000 mf/ml (OR 0.35, *P* = 0.001 and OR 0.46, *P* = 0.021, respectively).

**Table 4:** Binomial logistic regression for thick blood smear pairs with an MFD_2_ relative increase and decrease beyond the upper quartile

Characteristic	MFD_2_ relative increase beyond Q3^b^						MFD_2_ relative decrease beyond Q3^b^					
	Saturated model (AIC 832.21)			Final model^a^ (AIC 810.07)			Saturated model (AIC 590.2)			Final model^a^ (AIC 567.95)		
	OR	95% CI	*P*-value	OR	95% CI	*P* value	OR	95% CI	*P* value	OR	95% CI	*P* value
Intercept	0.28	0.12, 0.66	0.004	0.28	0.16, 0.49	< 0.001	0.17	0.05, 0.51	0.002	0.42	0.24, 0.73	0.003
Sex			0.710						0.751			
Male	1.00	–					1.00	–				
Female	0.92	0.58, 1.44	0.710				0.92	0.54, 1.54	0.751			
Age class (years)			0.700						0.776			
18–40	1.00	–					1.00	–				
41–50	0.86	0.50, 1.46	0.580				0.79	0.41, 1.52	0.489			
51–60	1.19	0.73, 1.94	0.481				1.12	0.65, 1.94	0.672			
61–70	1.04	0.59, 1.82	0.904				1.08	0.54, 2.13	0.832			
Weight class (kg)			0.159			0.058			0.359			
40–55	0.58	0.35, 0.93	0.026	0.56	0.36, 0.87	0.009	1.44	0.80, 2.65	0.229			
56–60	1.00	–		1.00	–		1.00	–				
61–65	0.87	0.49, 1.51	0.613	0.88	0.51, 1.51	0.652	1.48	0.75, 2.92	0.255			
66–85	0.81	0.47, 1.38	0.446	0.81	0.48, 1.35	0.421	1.97	0.88, 4.34	0.093			
Heart rate class (bpm)			0.445						0.454			
50–65	1.42	0.90, 2.26	0.135				1.66	0.89, 3.10	0.109			
66–75	1.00	–					1.00	–				
76–85	1.07	0.67, 1.70	0.789				1.29	0.70, 2.37	0.416			
86–115	0.97	0.51, 1.78	0.919				1.21	0.60, 2.44	0.595			
MAP class (mmHg)			0.426						0.004			0.006
55–80	0.78	0.46, 1.31	0.348				1.66	0.91, 3.04	0.101	1.45	0.82, 2.58	0.196
81–90	1.00	–					1.00	–		1.00	–	
91–100	0.79	0.48, 1.28	0.341				0.65	0.36, 1.18	0.159	0.61	0.34, 1.07	0.087
101–150	0.65	0.38, 1.09	0.105				0.55	0.28, 1.05	0.073	0.59	0.32, 1.06	0.081
Body temperature class			0.822						0.160			
34.3–36.3 °C	1.21	0.71, 2.08	0.488				1.16	0.59, 2.33	0.667			
36.4–36.5 °C	1.00	–					1.00	–				
36.6–36.7 °C	1.30	0.74, 2.34	0.365				1.39	0.68, 2.88	0.371			
36.8–37.8 °C	1.14	0.66, 1.98	0.646				2.04	1.01, 4.22	0.049			
MFD_1_ class (mf/ml)			< 0.001			< 0.001			< 0.001			< 0.001
1–1000	2.53	1.53, 4.26	< 0.001	2.39	1.48, 3.93	< 0.001	1.77	0.99, 3.20	0.057	1.72	1.01, 2.97	0.049
1001–3000	1.00	–		1.00	–		1.00	–		1.00	–	
3001–9000	1.11	0.64, 1.94	0.714	1.03	0.61, 1.77	0.903	0.61	0.32, 1.17	0.142	0.61	0.32, 1.12	0.112
9001–85,000	0.36	0.18, 0.69	0.002	0.35	0.18, 0.65	0.001	0.51	0.25, 1.01	0.057	0.46	0.23, 0.88	0.021
Microscopist			0.382						0.480			
Microscopist 1	1.00	–					1.00	–				
Microscopist 2	0.86	0.60, 1.21	0.383				1.16	0.76, 1.78	0.480			
Collection time (hour of day)			0.113			0.059			0.670			
10:00–10:59 a.m.	1.65	1.04, 2.62	0.032	1.69	1.10, 2.62	0.017	0.98	0.53, 1.78	0.937			
11:00–11:59 a.m.	1.00	–		1.00	–		1.00	–				
12:00–12:59 p.m.	1.09	0.66, 1.77	0.734	1.03	0.64, 1.64	0.912	1.13	0.66, 1.93	0.645			
1:00–1:59 p.m.	0.88	0.42, 1.76	0.723	0.91	0.44, 1.80	0.791	0.55	0.15, 1.63	0.319			
Collection time-point, day of study^c^			0.978						0.590			
D-5	1.00	–					1.00	–				
D2	0.96	0.62, 1.47	0.837				0.80	0.48, 1.33	0.388			
D7	0.99	0.64, 1.52	0.949				1.03	0.60, 1.75	0.928			

*AIC* Akaike information criterion, *bpm* beats per minute, *CI* confidence interval, *mf* microfilariae, *MFD*_1_, *MFD*_2_ microfilarial density of two thick blood smears generated from two successive blood samples

^a^Final model was selected by a procedure of AIC-based descending selection

^b^Upper quartile

^c^Blood samples were collected 5 days before levamisole intake (D-5) and at each follow-up visit (2, 7 and 30 days after treatment [D2, D7, D30, respectively﻿])

Considering MFD_2_ increase, TBSs generated from blood samples collected from subjects weighing 40–55 kg exhibited less variation (OR 0.56, *P* = 0.009; Table 4, final model), while samples collected between 10:00 and 10:59 a.m. yielded more change (OR 1.69, *P* = 0.017; Table 4, final model).

Considering MFD_2_ decrease, TBSs generated from blood samples collected from subjects belonging to the lowest MAP classes (55–80 mmHg) appeared to exhibit more variability (OR 1.45, *P* = 0.196; Table 4, final model) than higher MAP classes (101–150 mmHg; OR 0.59, *P* = 0.081, final model; Table 4), reaching overall significance (Wald’s test, *P* = 0.006) although failing to reach individual statistical significance.

Finally, the model analyzing TBS pairs with an MFD_2_ class increase (classes proposed by a loiasis treatment protocol [4]) showed that TBSs sampled from subjects with lower MFD_1_, lower MAP and sampled at an earlier hour in the day were more subject to MFD_2_ category changes, similar to previous models (Table 5). However, an effect of heart rate and sample collection time-point (D-5, and D2 or D7 after levamisole treatment) was also observed. Samples collected on D2 were found to have fewer MFD_2_ class increases (OR 0.41, *P* = 0.001, final model; Table 5), while TBSs generated from blood collected from subjects with a heart rate between 50–65 bpm and 76–85 bpm displayed more MFD_2_ class increases (OR 1.87, *P* = 0.026 and OR 2.04, *P* = 0.009, respectively; Table 5, final model) than those collected from subjects with a hear rate of 66–75 bpm.

**Table 5:** Binomial logistic regression studying thick blood smear pairs with an increase in MFD_2_ class (classes proposed by a loiasis treatment protocol [4])

Characteristic	Saturated model (AIC 602.4)			Final model^a^ (AIC 592.2)		
	OR	95% CI	*P* value	OR	95% CI	*P* value
Intercept	0.56	0.21, 1.52	0.259	0.51	0.24, 1.09	0.085
Sex			0.475			
Male	1.00	–				
Female	0.81	0.46, 1.43	0.477			
Age class (years)			0.226			
18–40	1.00	–				
41–50	1.86	1.03, 3.38	0.040			
51–60	1.23	0.67, 2.28	0.505			
61–70	1.37	0.66, 2.81	0.400			
Weight class (kg)			0.631			
40–55	0.74	0.42, 1.29	0.287			
56–60	1.00	–				
61–65	0.69	0.34, 1.37	0.296			
66–85	0.78	0.39, 1.51	0.460			
Heart rate class (bpm)			0.001			0.001
50–65	1.95	1.09, 3.51	0.025	1.87	1.08, 3.27	0.026
66–75	1.00	–		1.00	–	
76–85	2.30	1.32, 4.05	0.003	2.04	1.20, 3.52	0.009
86–115	0.68	0.28, 1.54	0.369	0.58	0.25, 1.28	0.194
MAP class (mmHg)			0.052			0.063
55–80	1.11	0.61, 2.03	0.723	1.13	0.63, 2.01	0.677
81–90	1.00	–		1.00	–	
91–100	0.48	0.25, 0.90	0.023	0.50	0.27, 0.92	0.027
101–150	0.73	0.39, 1.37	0.329	0.80	0.44, 1.44	0.464
Body temperature class			0.622			
34.3–36.3 °C	0.87	0.48, 1.61	0.665			
36.4–36.5 °C	1.00	–				
36.6–36.7 °C	0.93	0.50, 1.75	0.825			
36.8–37.8 °C	0.66	0.34, 1.28	0.222			
MFD_1_ class (mf/ml)			< 0.001			< 0.001
1–1000	0.03	0.00, 0.09	< 0.001	0.02	0.00, 0.08	< 0.001
1001–3000	1.00	–		1.00	–	
3001–9000	2.59	1.50, 4.55	< 0.001	2.33	1.40, 3.94	0.001
9001–85,000	0.50	0.27, 0.90	0.023	0.56	0.32, 1.00	0.049
Microscopist			0.078			0.097
Microscopist 1	1.00	–		1.00	–	
Microscopist 2	0.69	0.45, 1.04	0.079	0.71	0.46, 1.06	0.098
*Collection time (hour of day)*			< 0.001			< 0.001
10:00–10:59 a.m.	1.32	0.79, 2.21	0.286	1.35	0.84, 2.19	0.213
11:00–11:59 a.m.	1.00	–		1.00	–	
12:00–12:59 p.m.	0.45	0.24, 0.82	0.010	0.47	0.26, 0.84	0.012
1:00–1:59 p.m.	0.23	0.05, 0.74	0.026	0.20	0.04, 0.61	0.012
Collection time-point, day of study^b^			0.006			0.004
D-5	1.00	–		1.00	–	
D2	0.41	0.24, 0.71	0.002	0.41	0.24, 0.69	0.001
D7	0.70	0.42, 1.17	0.178	0.67	0.41, 1.10	0.113

MFD_2_ classes were based on a previously published loiasis treatment protocol [4])

*AIC* Akaike information criterion, *bpm* beats per minute, *CI* confidence interval, *mf* microfilariae, *MFD*_*1*_, *MFD*_*2*_ microfilarial density of two thick blood smears generated from two successive blood samples

^a^Final model was selected by a procedure of Akaike Information Criterion-based descending selection

^b^Blood samples were collected 5 days before levamisole intake (D-5) and at each follow-up visit (2, 7 and 30 days after treatment [D2, D7, D30, respectively﻿])

### Changes in MFD class

A comparison of the distribution of MFD_1_ and MFD_2_ classes according to a loiasis treatment protocol [4] is shown in Table 6. Between MFD_1_ and MFD_2_, 204 TBS pairs (11%) increased by one class and six TBS pairs (0.3%) increased by two classes, while 121 TBS pairs (6%) decreased by one class and two TBS pairs (0.1%) decreased by two classes, yielding a total of 333 (17.3%) class changes. Most variation was seen in groups 2001–8000 mf/ml and 8001–30,000 mf/ml, where between 25 and 30% of MFD_2_ showed a class change compared to MFD_1_. Up to 9.4% of MFD_1_ in the 8001–30,000 mf/ml class had an MFD_2_ class > 30,000 mf/ml.

**Table 6:** Transition matrix comparing MFD_1_ and MFD_2_ classes

Classes	MFD class (mf/ml)	MFD_2_				Overall
		0–2000 mf/ml	2001–8000 mf/ml	8001–30000 mf/ml	> 30,000 mf/ml	–
MFD_1_	0–2000	729 (92%)	62 (7.8%)	4 (0.5%)	0 (0%)	795 (100%)
	2001–8000	47 (9.1%)	364 (70%)	104 (20%)	2 (0.4%)	517 (100%)
	8001–30,000	2 (0.5%)	60 (15%)	305 (75%)	38 (9.4%)	405 (100%)
	> 30,000	0 (0%)	0 (0%)	14 (13%)	95 (87%)	109 (100%)

MFD_1_ and MFD_2_ classes were based on a previously published loiasis treatment protocol [4])

The left column displays MFD_1_ categories, and the top row displays MFD_2_ categories. Percentages are computed in rows, with the right overall column displaying 100% of the sample

*mf* Microfilariae, *MFD*_*1*_, *MFD*_*2*_ microfilarial density of two thick blood smears generated from two successive blood samples

Among the six TBSs with a two-class increase, MFD_1_ and MFD_2_ were 1820 and 11,120 mf/ml, 1840 and 12,180 mf/ml, 1640 and 9780 mf/ml, 1260 and 10,880 mf/ml, 7020 and 47,300 mf/ml and 5100 and 43,360 mf/mL, respectively (Fig. 1).

We also compared the distribution of MFD_1_ and MFD_2_ arithmetic means with MFD_1_ and MFD_2_ (Table 7). As expected, the MFD mean lead to fewer class changes, although 6.4% of MFD means in the 8001–30000 mf/ml class had an MFD_1_ (2.3%) or MFD_2_ (4.1%) > 30,000 mf/ml.

**Table 7:** Transition matrix comparing (MFD_1_ + MFD_2_)/2 with MFD_1_ and MFD_2_ classes

	MFD class (mf/ml)	MFD_1_				MFD_2_				Overall
		0–2000	2001–8000	8001–30000	> 30,000	0–2000	2001–8000	8001–30000	> 30,000	–
(MFD_1_ + MFD_2_)/2	0–2000	755 (97%)	21 (2.7%)	0 (0%)	0 (0%)	750 (97%)	26 (3.4%)	0 (0%)	0 (0%)	776 (100%)
	2001–8000	40 (8.1%)	422 (85%)	33 (6.7%)	0 (0%)	27 (5.5%)	432 (87%)	36 (7.3%)	0 (0%)	495 (100%)
	8001–30,000	0 (0%)	74 (17%)	350 (81%)	10 (2.3%)	1 (0.2%)	28 (6.5%)	387 (89%)	18 (4.1%)	434 (100%)
	> 30,000	0 (0%)	0 (0%)	22 (18%)	99 (82%)	0 (0%)	0 (0%)	4 (3.3%)	117 (97%)	121 (100%)

MFD_1_ and MFD_2_ classes were based on a previously published loiasis treatment protocol [4])

The left column displays (MFD_1_ + MFD_2_)/2 categories, and the top row displays MFD_1_ and MFD_2_ categories. Percentages are computed in rows, with the right overall column displaying 100% of the sample

*mf* Microfilariae, *MFD*_*1*_, *MFD*_*2*_ microfilarial density of two thick blood smears generated from two successive blood samples

## Discussion

Overall, MFD_2_ were higher than MFD_1_ with a mean ratio of 1.28, indicating that on average, MFD_2_ was 28% higher than MFD_1_. This observation was mainly driven by the fact that 63% (1145/1826) of TBSs exhibited an MFD_2_ increase, while only 37% (681/1826) exhibited a decrease.

Since Kershaw et al.’s preliminary work in 1950 [8], studies investigating the variability of *L. loa* microfilarial counts in successive blood smears from the same fingerprick have been lacking, although some data exist for other filarial species. Marked individual MFD variations have been observed for *Wuchereria bancrofti*, the major cause of lymphatic filariasis, although overall MFD averages were found not to significantly differ between successive TBSs [11, 12]. In contrast to our results, the MFD variability of successive TBSs from a single puncture appeared to be equivalent to that of samples from independent punctures [13]. For *Dirofilaria* sp., whose mf also circulate in the blood, Hawking et al. reported considerable MFD differences in consecutive blood samples collected from primates [14], with the first TBS containing more mf than subsequent TBSs, a trend which was also reported by the same author in a subsequent publication [15]. This heterogeneity in MFD was also studied by experimental infection of vectors. During a human blood meal, the number of ingested *W. bancrofti* mf by *Aedes* or *Culex* mosquitoes followed a binomial negative distribution [16]. The authors hypothesized that mf could form “waiting queues” in blood capillaries, constituting an uneven distribution that would explain MFD variability [17].

However, that uneven repartition is likely stochastic and cannot explain the trend we observed with *L. loa* toward an increase between MFD_1_ and MFD_2_. This result is difficult to explain, but it could suggest that mf repartition in capillaries is not uneven and that applying a second pressure to a punctured finger to sustain the blood flow can mobilize a vascular reservoir of mf that the first pressure is unable to.

The MFD relative change was 61% and − 29% for MFD_2_ increase and decrease, respectively. These variations drove a total of 333 (17.4%) MFD category changes according to a previously reported loiasis treatment protocol [4], reaching up to 30% in some subgroups (subjects with MFD_1_ between 2001 and 8000 mf/ml). Among these 333 category changes, 210 (11.3%) TBSs exhibited an MFD_2_ class increase. These variations may impact loiasis treatment allocation, which is based on patients’ MFD classes, and might increase the risk of post-treatment SAEs. A patient may be allocated IVM based on an MFD_1_ < 30,000 mf/ml, whereas its MFD_2_ far exceeds 30,000 mf/ml, a clinical situation which would strongly advise against the use of IVM due to the risk of post-treatment SAE in this population. Notably, this specific scenario occurred in 40 TBS pairs in our study, representing up to 9.4% of TBS pairs with an MFD_1_ between 8001 and 30,000 mf/ml.

Despite growing interest in loiasis and subsequent development of clinical trials due to its observed related mortality, there are currently no clinical guidelines supporting the collection and analysis of one or two TBSs. On the basis of MFD_2_ being on average higher than MFD_1_ in our study, we propose that two 50-µl blood samples be collected systematically for generating TBSs, with both MFD_1_ and MFD_2_ being assessed. Treatment allocation may then be based on the highest of the two MFDs, which would be the safest option. Treatment may also be based on the arithmetic mean of both MFDs, a strategy regularly used in clinical trials on loiasis [9, 10]. However, 6.4% of MFD means in the 8001–30000 mf/ml class had an MFD_1_ or MFD_2_ that exceeded to 30,000 mf/ml in our study, which might be an issue depending on the protocol of the trial. As MFD thresholds of post-treatment SAE risk were originally solely based on the evaluation of a single TBS [4], we cannot be sure that the analysis of two TBSs would effectively increase safety regarding possible post-treatment SAEs. However, given this marked MFD variability, MFD_2_ assessments may be useful to attempt to explain cases of SAEs occurring with a MFD_1_ below the risk threshold.

Our attempt to identify explanatory variables of the difference between MFD_1_ and MFD_2_ by studying the characteristics of individuals with extreme differences identified several candidate factors. As expected, patients with an MFD_1_ < 1000 mf/ml had more variability, given that when MFDs are low, only a small difference has an important impact on the relative change computation.

Subjects whose TBSs showed an MFD change > 2 SD had significantly lower MAP. After adjustment by binomial logistic regression, this trend remained significant in TBSs with an MFD_2_ decrease and class increase. The manner in which MAP could impact MFD variation is unclear. It is known that hypotension regimens can lead to blood capillary closure. When intravascular pressure drops below a “critical closing pressure,” it can no longer compensate for the surrounding tissue pressure, which ultimately leads to capillary collapse [18]. Moreover, sympathetic nervous system activation induces vasoconstriction that further accelerates cutaneous capillary closure, to maintain the perfusion pressure of vital organs [19]. Given that the diameter of *L. loa* mf (5–7 µm) is close to that of capillaries, we propose that this situation might substantially alter their circulation, leading to an increased change in MFD.

Finally, there was no trend in our transition evaluation of MFD relative change, which was not in the same direction at different time-points for a given person.

Our work has a number of limitations. The data originate from a clinical trial that was not designed for the purpose of this analysis. We cannot exclude an impact of the trial’s treatment (levamisole) on the conclusions of our study, although a differential treatment effect on only one of the TBSs is unlikely. While we accounted for inter-operator variability by computing MFD change from the results of a single microscopist, intra-operator variability was not considered and might be a confounding factor. Future studies are needed to further document successive TBS variability, including dedicated studies with larger samples and three or more successive TBSs, to determine how variability behaves across multiple samples.

## Conclusions

To our knowledge, this is the first work describing MFD variability of successive TBS from a single finger puncture in more than 200 subjects with loiasis. We observed a trend toward an increase in MFD_2_ between samples at a magnitude that may impact treatment choice. Our results suggest that systematically sampling at least two successive TBSs could allow a better MFD assessment and proper treatment allocation to prevent SAEs. If future works confirm our results, the potential explanatory variables identified here might ultimately help select sampling conditions that could minimize variability in successive TBSs.

## Supplementary Information

Supplementary Material 1.

## Data Availability

Data supporting the conclusions of this article are included within the article. The datasets used and/or analyzed during the present study are available from the corresponding author upon reasonable request.
