# Supplementary material for: Variability of Loa loa microfilarial counts in successive blood smears and its potential implication in drug-related serious adverse events
Source: Parasit Vectors. 2024 Nov 8;17:457. doi: 10.1186/s13071-024-06494-0 (PMC11549846; doi:10.1186/s13071-024-06494-0)
Supplement: Supplementary file 1 — Supplementary Material 1. [file 13071_2024_6494_MOESM1_ESM.docx]

# Supplementary information

# Table S1. Comparisons of MFD_1_ and MFD_2_

| Characteristic | MFD_1_, GM^1^ (range) | MFD_2_, GM^1^ (range) | *P* value^2^ | MFD Ratio^3^,  mean (SD) |
| --- | --- | --- | --- | --- |
| Total, N = 1826 | 2335 (20–80580) | 2570 (20–87420) | < 0.001 | 1.28 (0.84) |
| Sex |  |  |  |  |
| Males, N = 1277 | 2626 (20–77260) | 2913 (20–85080) | < 0.001 | 1.28 (0.80) |
| Females, N = 549 | 1777 (20–80580) | 1922 (20–87420) | < 0.001 | 1.26 (0.91) |
| Age class (years old) |  |  |  |  |
| 18-40, N = 554 | 2097 (20–73060) | 2288 (20–76100) | < 0.001 | 1.25 (0.75) |
| 41-50, N = 399 | 4104 (20–80580) | 4538 (20–79860) | < 0.001 | 1.24 (0.63) |
| 51-60, N = 526 | 2425 (20–77260) | 2700 (20–85080) | < 0.001 | 1.31 (0.91) |
| 61-70, N = 347 | 1369 (20–64000) | 1494 (20–87420) | < 0.001 | 1.32 (1.04) |
| Weight class (kg) |  |  |  |  |
| 40-55, N = 749 | 2197 (20–80580) | 2307 (20–87420) | < 0.001 | 1.22 (0.88) |
| 56-60, N = 510 | 2573 (20–54160) | 2935 (20–79860) | < 0.001 | 1.31 (0.72) |
| 61-65, N = 290 | 2803 (40–77260) | 2953 (20–85080) | < 0.001 | 1.23 (0.86) |
| 66-85, N = 277 | 1900 (20–73060) | 2332 (20–76100) | < 0.001 | 1.42 (0.86) |
| Heart rate class (bpm) |  |  |  |  |
| 50-65, N = 339 | 2379 (20–72240) | 2603 (20–79860) | < 0.001 | 1.27 (0.82) |
| 66-75, N = 412 | 2259 (20–77260) | 2456 (20–85080) | < 0.001 | 1.22 (0.66) |
| 76-85, N = 376 | 2185 (20–64000) | 2299 (20–75460) | < 0.001 | 1.26 (1.07) |
| 86-115, N = 214 | 2797 (40–73000) | 2735 (20–71620) | 0.456 | 1.13 (0.69) |
| MAP class (mmHg) |  |  |  |  |
| 55-80, N = 277 | 2200 (20–71520) | 2297 (20–54040) | < 0.001 | 1.20 (0.67) |
| 81-90, N = 359 | 2164 (20–64100) | 2277 (20–80520) | < 0.001 | 1.28 (1.06) |
| 91-100, N = 356 | 2117 (20–72240) | 2281 (20–75460) | 0.001 | 1.26 (0.94) |
| 101-150, N = 353 | 2976 (20–77260) | 3158 (20–85080) | 0.001 | 1.17 (0.54) |
| Body temperature class (°C) | |  |  |  |
| 34.3 – 36.3, N = 426 | 1746 (20–64000) | 1816 (20–70220) | < 0.001 | 1.24 (1.02) |
| 36.4 – 36.5, N = 261 | 3214 (20–72240) | 3489 (20–80520) | < 0.001 | 1.20 (0.57) |
| 36.6 – 36.7, N = 331 | 3808 (40–77260) | 4001 (40–71620) | < 0.001 | 1.21 (0.72) |
| 36.8 – 37.8, N = 327 | 1645 (20–76020) | 1767 (20–85080) | < 0.001 | 1.27 (0.87) |
| MFD_1_ class (mf/mL) |  |  |  |  |
| 1-1000, N = 582 | 291 (20–1000) | 333 (20–4220) | < 0.001 | 1.44 (1.13) |
| 1001-3000, N = 367 | 1782 (1020–3000) | 1928 (280–12180) | < 0.001 | 1.23 (0.79) |
| 3001-9000, N = 448 | 5715 (3020–9000) | 6567 (200–47300) | < 0.001 | 1.27 (0.70) |
| 9001-85000, N = 429 | 19,437 (9020–80580) | 19,768 (920–87420) | < 0.001 | 1.10 (0.39) |
| Microscopist |  |  |  |  |
| Microscopist 1, N = 917 | 2232 (20–80580) | 2506 (20–85080) | < 0.001 | 1.32 (0.92) |
| Microscopist 2, N = 909 | 2444 (20–77260) | 2636 (20–87420) | < 0.001 | 1.24 (0.74) |
| Collection hours |  |  |  |  |
| 10:00-10:59 am, N = 494 | 3428 (20–73060) | 4200 (20–79860) | < 0.001 | 1.43 (1.00) |
| 11:00-11:59 am, N = 731 | 1963 (20–80580) | 2001 (20–87420) | < 0.001 | 1.18 (0.80) |
| 12:00-12:59 pm, N = 467 | 2343 (20–41740) | 2550 (20–58820) | < 0.001 | 1.23 (0.66) |
| 1:00-1:59 pm, N = 134 | 1441 (20–27440) | 1695 (40–22760) | < 0.001 | 1.37 (0.81) |
| Collection time point |  |  |  |  |
| D-5, N = 470 | 2217 (20–77260) | 2345 (20–80520) | 0.001 | 1.25 (0.96) |
| D2, N = 455 | 2461 (20–64100) | 2523 (20–75460) | 0.012 | 1.16 (0.61) |
| D7, N = 443 | 2391 (40–76020) | 2654 (20–85080) | < 0.001 | 1.30 (0.91) |
| D30, N = 458 | 2284 (20–80580) | 2789 (20–87420) | < 0.001 | 1.40 (0.79) |

^1^ Geometric mean ^2^ Wilcoxon signed-rank test

^3^The last column displays the mean ratio, defined as MFD_2_/MFD_1_.

**Table S2. MFD_1_ and MFD_2_ transition evaluation of D-5 with D2, D7 and D30, at an individual-level.**

|  |  |  | D2 | | | D7 | | | D30 | | |
| --- | --- | --- | --- | --- | --- | --- | --- | --- | --- | --- | --- |
|  |  |  | Stable | MFD_2_ < MFD_1_ | MFD_2_ > MFD_1_ | Stable | MFD_2_ < MFD_1_ | MFD_2_ > MFD_1_ | Stable | MFD_2_ < MFD_1_ | MFD_2_ > MFD_1_ |
| D-5 | Stable | Microscopist 1 | 33 (43.4) | 18 (23.7) | 25 (32.9) | 24 (32.9) | 16 (21.9) | 33 (45.2) | 26 (34.7) | 6 (8.0) | 43 (57.3) |
|  | MFD_2_ < MFD_1_ |  | 27 (40.9) | 19 (28/8) | 20 (30.3) | 22 (35.5) | 20 (32.3) | 20 (32.3) | 21 (33.9) | 15 (24.2) | 26 (41.9) |
|  | MFD_2_ > MFD_1_ |  | 25 (28.7) | 24 (27.6) | 38 (43.7) | 28 (32.6) | 13 (15.1) | 45 (52.3) | 22 (24.2) | 16 (17.6) | 53 (58.2) |
|  | Kappa |  | 0.08 (P = 0.037) | | | 0.09 (P = 0.033) | | | 0.09 (P = 0.033) | | |
|  | McNemar test |  | 0.356 | | | 0.919 | | | 0.532 | | |
|  | Stable | Microscopist 2 | 34 (41.5) | 25 (30.5) | 23 (28.0) | 32 (41.6) | 9 (11.7) | 36 (46.7) | 25 (30.1) | 15 (18.1) | 43 (51.8) |
|  | MFD_2_ < MFD_1_ |  | 24 (41.4) | 14 (24.1) | 20 (34.5) | 17 (29.8) | 16 (28.1) | 24 (42.1) | 20 (36.4) | 6 (10.9) | 29 (52.7) |
|  | MFD_2_ > MFD_1_ |  | 31 (37.4) | 22 (26.5) | 30 (36.1) | 29 (34.5) | 13 (15.5) | 42 (50.0) | 30 (34.9) | 13 (15.1) | 43 (50.0) |
|  | Kappa |  | 0.01 (P = 0.375) | | | 0.099 (P = 0.026) | | | -0.04 (P = 0.822) | | |
|  | McNemar test |  | 0.490 | | | 0.917 | | | 0.441 | | |

**Table S3. MFD_1_ and MFD_2_ transition evaluation of D2 with D7 and D30, at an individual-level.**

|  |  |  | D7 | | | D30 | | |
| --- | --- | --- | --- | --- | --- | --- | --- | --- |
|  |  |  | Stable | MFD_2_ < MFD_1_ | MFD_2_ > MFD_1_ | Stable | MFD_2_ < MFD_1_ | MFD_2_ > MFD_1_ |
| D2 | Stable | Microscopist 1 | 31 (39.7) | 13 (16.7) | 34 (43.6) | 24 (29.3) | 11 (13.4) | 47 (57.3) |
|  | MFD_2_ < MFD_1_ |  | 20 (33.3) | 16 (26.7) | 24 (40.0) | 19 (32.8) | 9 (15.5) | 30 (51.7) |
|  | MFD_2_ > MFD_1_ |  | 23 (27.8) | 19 (23.8) | 38 (47.5) | 25 (30.1) | 15 (18.1) | 43 (41.8) |
|  | Kappa |  | 0.071 (P = 0.071) | | | -0.02 (P = 0.660) | | |
|  | McNemar test |  | 0.673 | | | 0.166 | | |
|  | Stable | Microscopist 2 | 30 (34.5) | 12 (13.8) | 45 (51.7) | 26 (30.2) | 11 (12.8) | 49 (57.0) |
|  | MFD_2_ < MFD_1_ |  | 20 (33.9) | 13 (22.0) | 26 (44.1) | 19 (31.7) | 13 (21.7) | 28 (46.7) |
|  | MFD_2_ > MFD_1_ |  | 27 (37.5) | 14 (19.4) | 31 (43.1) | 29 (39.2) | 8 (10.8) | 37 (50.0) |
|  | Kappa |  | -0.007 (P = 0.557) | | | < 0.001 (P = 0.500) | | |
|  | McNemar test |  | 0.327 | | | 0.248 | | |

**Table S4. MFD_1_ and MFD_2_ transition evaluation of D7 with D30, at an individual-level.**

|  |  |  | D30 | | |
| --- | --- | --- | --- | --- | --- |
|  |  |  | Stable | MFD_2_ < MFD_1_ | MFD_2_ > MFD_1_ |
| D7 | Stable | Microscopist 1 | 17 (23.0) | 13 (17.6) | 44 (59.5) |
|  | MFD_2_ < MFD_1_ |  | 16 (34.0) | 9 (19.2) | 22 (46.8) |
|  | MFD_2_ > MFD_1_ |  | 31 (32.6) | 12 (12.6) | 52 (54.7) |
|  | Kappa |  | -0.02 (P = 0.687) | | |
|  | McNemar test |  | 0.327 | | |
|  | Stable | Microscopist 2 | 22 (28.2) | 8 (10.3) | 48 (61.5) |
|  | MFD_2_ < MFD_1_ |  | 18 (48.7) | 7 (18.9) | 12 (32.4) |
|  | MFD_2_ > MFD_1_ |  | 34 (33.3) | 18 (17.7) | 50 (49.0) |
|  | Kappa |  | -0.04 (P = 0.768) | | |
|  | McNemar test |  | 0.700 | | |
